# Genome-based characterization of AHPND and non-AHPND *Vibrio campbellii* isolates from Republic of Korea

**DOI:** 10.3389/fmicb.2026.1724818

**Published:** 2026-01-26

**Authors:** So-Young Lee, Hee-Jae Choi, Seonhyun Lee, Jaeyoung Choi, Jun Soung Kwak, Yue Jai Kang, Sung-Chul Hong

**Affiliations:** 1 Department of Food Science and Biotechnology, Kunsan National University, Gunsan, Republic of Korea; 2 Jeju Regional Office, National Fishery Products Quality Management Service, Jeju, Republic of Korea; 3 Department of Oriental Medicine Biotechnology, College of Life Sciences, Kyung Hee University, Yongin, Republic of Korea; 4 Department of Convergent Biotechnology and Advanced Materials Science, Kyung Hee University, Yongin, Republic of Korea; 5 BK21 Interdisciplinary Program in IT-Bio Convergence System, Kyung Hee University, Yongin, Republic of Korea; 6 Department of Aquatic Life Medicine, Kongju National University, Gongju, Republic of Korea; 7 Department of Aquatic Life Medicine, Kunsan National University, Gunsan, Republic of Korea; 8 Fisheries Science Institute, Kunsan National University, Gunsan, Republic of Korea

**Keywords:** acute hepatopancreatic necrosis disease, comparative genomics, genomic features, shrimp aquaculture, *Vibrio campbellii*, virulence factors

## Abstract

With mounting evidence that *Vibrio campbellii* can act as a causative agent, acute hepatopancreatic necrosis disease (AHPND) represents a serious threat to global shrimp aquaculture. In this study, we present a comparative genomic analysis of 101 *V. campbellii* strains, including the recently isolated pathogenic and non-pathogenic strains, *V. campbellii* HJ-2023 and *V. campbellii* HJ-2023n, from the Republic of Korea. Whole-genome sequencing revealed that the pathogenic strain harbors three plasmids and carries the canonical AHPND toxin genes *pirA* and *pirB*, along with an expanded repertoire of virulence and secretion system genes. Pan-genome and insertion sequence analyses showed that pathogenic strains tend to cluster based on shared mobile genetic elements, particularly transposases located near toxin genes, underscoring the role of horizontal gene transfer in virulence acquisition. Although all strains displayed a broad distribution of antibiotic-resistance genes, pathogenicity did not consistently correlate with their presence. Similarly, carbohydrate-active enzyme (CAZyme) profiles were largely conserved, although certain enzymes, such as chitinases, may contribute accessory functions in host invasion. Notably, the AHPND-associated *V. campbellii* HJ-2023 strain contained multiple copies of key *T6SS* and *T1SS* genes, suggesting an increased potential for toxin delivery. These findings suggest that pathogenic potential in *V. campbellii* likely arises not only from the presence of toxins but also from the complex interplay of mobile elements, secretion systems, and genomic architecture. This study provides an essential genomic framework for understanding the emergence of AHPND in *V. campbellii* and offers insights to enhance molecular diagnostics, strengthen biosecurity, and improve disease control strategies in shrimp aquaculture.

## 1 Introduction

The ongoing crisis of global climate change has heightened the importance of securing sustainable food resources worldwide (Costello et al., 2020). As marine environments cover nearly 70% of the Earth’s surface, marine resources have become increasingly vital to global food security (Golden et al., 2021). Within this context, shrimp aquaculture represents one of the most rapidly expanding sectors of marine food production, experiencing remarkable growth over the past three decades. For instance, the area devoted to shrimp farming in the Gulf of California, northwest Mexico, expanded by more than 1,100% between 1993 and 2021 (González-Rivas and Tapia-Silva, 2023). However, despite this substantial growth, outbreaks of acute hepatopancreatic necrosis disease (AHPND) have recently emerged as a devastating threat, causing severe losses in shrimp aquaculture (Kumar et al., 2021).

First reported in China in 2009, AHPND rapidly spread to Vietnam (2010), Malaysia (2011), Thailand (2012), Mexico (2013), the Philippines (2015), South America (2016), and most recently, the Republic of Korea (2023) (Abu Hassan et al., 2025; Choi et al., 2024). The disease has had its greatest impact in Asia, which produces approximately 70% of the world’s shrimp supply, leading to estimated losses exceeding one billion US dollars (Flegel, 2012; Powell et al., 2015). The high mortality rates associated with AHPND, also known as early mortality syndrome, have severely undermined shrimp production (Thadtapong et al., 2020; Sun and Chen, 2014).

Early studies on AHPND identified *Vibrio parahaemolyticus* as the causative bacterial agent. Pathogenic *V. parahaemolyticus* strains typically harbor a specific plasmid (pVA1 type) that carries two toxin genes, *pirA* and *pirB*. These genes encode a binary toxin responsible for the hepatopancreatic damage characteristic of AHPND (Xiao et al., 2017). Furthermore, the high homology between *pirA* and *pirB* toxins and those produced by the insect pathogen *Photorhabdus* spp. suggests that horizontal gene transfer (HGT) events introduced these genes into *Vibrio* species (Fu et al., 2020).

Although early studies largely identified *V. parahaemolyticus* as the sole causative agent of AHPND, recent findings—particularly from Asian regions such as the Republic of Korea—have expanded this to include multiple *Vibrio* species, including *Vibrio campbellii*, *Vibrio owensii*, and *Vibrio punensis* (Choi et al., 2024). These species also carry pVA1-type plasmids encoding toxin genes, thereby broadening the spectrum of pathogenicity. Acquired through HGT, these plasmids contain a complex mobile genetic element, transposon Tn6264, which comprises the *pirA* and *pirB* toxin clusters flanked by ISVal1 insertion sequences (ISs) (McColl and Sunarto, 2015; Dong et al., 2017). These elements facilitate conjugation-mediated interspecies transfer, adding to the complexity of pathogen evolution and posing significant challenges for disease management (Lee et al., 2015).

Two strains previously isolated from shrimp farms in Taean-gun, Chungcheongnam-do, Republic of Korea, were identified as follows: HJ-2023 as pathogenic and HJ-2023n as non-pathogenic (Choi et al., 2024). Based on 16S rRNA gene sequencing, these strains shared more than 99% similarity with *V. parahaemolyticus*; however, phylogenetic analysis using *rpoD* sequences placed them in distinct clusters, limiting the resolution of their phylogenetic classification (Choi et al., 2024).

Consequently, investigating the genomic background and diversity of plasmid-mediated pathogenicity acquisition is essential. Although the presence of plasmids is a genomic marker distinguishing pathogenic from non-pathogenic strains (Lee et al., 2015), other genomic elements—such as transposable elements that associated with plasmid stability (Xiao et al., 2017), putative virulence factors implicated in host-pathogen interactions (Lightner et al., 2015), and metabolic enzymes that support environmental survival (Dong et al., 2017)—likely influence pathogenicity and transmission dynamics. Despite the urgent need for comprehensive genome-wide comparisons between pathogenic and non-pathogenic strains, most studies to date have focused primarily on AHPND plasmids and their associated toxin genes.

To address these gaps, this study conducted an integrated comparative genomic analysis to elucidate the evolutionary mechanisms underlying plasmid acquisition and pathogenicity at the whole-genome level. Using pan-genomic approaches, we compared shared and strain-specific gene clusters, identified key pathogenicity-associated genes, characterized transposable elements, and predicted secreted proteins. Leveraging two previously characterized *V. campbellii* strains, this comprehensive genomic investigation aimed to identify potential genomic features associated with AHPND pathogenicity. Ultimately, the study aimed to advance understanding of plasmid-mediated pathogenicity mechanisms, provide insights for future strategies in disease prevention and control, and contribute to the broader field of molecular epidemiology.

## 2 Materials and methods

### 2.1 Bacterial isolation and culture conditions

Shrimp samples (*Penaeus vannamei*) were randomly collected from aquaculture farms in Taean-gun, Chungcheongnam-do, Republic of Korea. Hepatopancreas tissues from five individuals were aseptically excised and streaked onto thiosulfate citrate bile salt sucrose agar (Difco, Franklin Lakes, NJ, United States). Following incubation at 27 °C for 48 h, individual colonies were subcultured. Species identification was confirmed by 16S rRNA sequencing. The AHPND-positive isolate was designated *V. campbellii* HJ-2023, whereas the AHPND-negative isolate was designated *V. campbellii* HJ-2023n.

### 2.2 Whole-genome sequencing and genome assembly

Genomic DNA was extracted using the DNeasy Blood & Tissue Kit (Qiagen, Germany) following the manufacturer’s protocol. Libraries were prepared with the Nextera XT DNA Library Prep Kit (Illumina) and sequenced on the Illumina NovaSeq 6000 and PacBio hybrid platforms. Raw reads were quality-filtered using Trimmomatic v0.39 (Bolger et al., 2014), and genome assembly was performed with the Hierarchical Genome Assembly Process (HGAP v3.0) (Chin et al., 2013). Annotation was conducted using Prokka v1.14.6 (Seemann, 2014), and chromosomal and plasmid components were predicted with geNomad v1.11.0 (Camargo et al., 2024) Genomic features, predicted genes, and biosynthetic gene cluster distributions were visualized in circular plots using Circos v0.69-9. Whole-genome-based taxonomic analysis was performed using Orthologous Average Nucleotide Identity (OrthoANI), with values calculated by the OrthoANI Tool v1.40 (Lee et al., 2016). To further validate the taxonomic classification, digital DNA–DNA hybridization (dDDH) values were determined using the Genome-to-Genome Distance Calculator (GGDC 3.0) web server (Meier-Kolthoff et al., 2013).

### 2.3 Genome collection and *pirA*/*pirB* gene screening

A total of 99 publicly available *V. campbellii* genome sequences were retrieved from the NCBI RefSeq database. The presence of *pirA* and *pirB* toxin genes was evaluated using BLASTP v2.16.0, with reference sequences obtained from GenBank (JBRBAD000000000 and JBRBAE000000000). Screening parameters were set to an *e*-value threshold of ≤1 × 10^−10^ and a minimum identity of 70%. Strains were classified as *pirA*/*pirB*-positive (*pirA*+/*pirB*+) or *pirA*/*pirB*-negative (*pirA*−/*pirB*−) based on detection status.

### 2.4 Pan-genome analysis and clustering analysis

Pan-genome clustering of the 101 *V. campbellii* strains was performed using PPanGGOLiN v2.1.0 (Gautreau et al., 2020). The resulting gene presence/absence matrix was compared with the distribution of *pirA* and *pirB* genes. Network-based visualization of gene co-occurrence was generated in Gephi v0.10.1 using the ForceAtlas2 layout algorithm (scaling = 8,000, gravity = 4.0, edge weight influence = 1.3) (Bastian et al., 2009). Nodes represented gene clusters, with coloring based on *pirA/pirB* status. Key hub genes were identified using degree thresholds (≥150 for AHPND-only and ≥100 for non-AHPND). Dimensionality reduction and classification analyses were performed with t-distributed stochastic neighbor embedding (t-SNE) and Information Gain methods in Orange3 v3.38.1 (Demšar et al., 2013). Metadata included strain country of origin and *pirA/pirB* classification.

### 2.5 Bioinformatics analyses

Virulence factor genes were identified using BLASTP searches against the Virulence Factor Database^1^ with a minimum identity of ≥70% and an *e*-value threshold of ≤1 × 10^−5^ (Zhou et al., 2025). Whole-proteome-based phylogenetic trees were constructed with CVTree v3.0 (Zuo and Hao, 2015). The insertion sequence (IS) elements were detected using ISEScan v1.7.2.3 (Xie and Tang, 2017), and secretion system gene clusters were annotated with DeepSecE v0.1.2 (Zhang et al., 2023). Antibiotic resistance genes were predicted using ABRicate v0.9.8 in combination with the ResFinder, Comprehensive Antibiotic Resistance Database (CARD), and NCBI databases (Zankari et al., 2012; Jia et al., 2017; Feldgarden et al., 2019). Carbohydrate-active enzymes (CAZymes) were predicted with dbCAN3 v4.1.4 (Zheng et al., 2023), and their distribution profiles were analyzed using the machine learning framework Orange v3.38.1 (Demšar et al., 2013). CAZyme profiles were modeled to classify *pirA/pirB*-positive and *pirA/pirB*-negative genomes using nine algorithms, and model performance was evaluated by stratified 10-fold cross-validation with key metrics (Supplementary Table S10). Presence/absence matrices were generated and compared among strains. Visualization, together with the CVTree-derived phylogeny, was performed using GraPhlAn v1.1.3 (Asnicar et al., 2015) or R v4.6.3 with the ggtree v3.14.0 package (Yu et al., 2017; R Core Team, 2025). Detailed software versions, command lines, database information, and parameter settings for all bioinformatic analyses are provided in Supplementary Text S1 to ensure reproducibility.

### 2.6 Statistical analyses

Statistical comparisons of genomic features (e.g., copy numbers of IS elements, Secretion Systems, ARGs, and CAZymes) between *pirA*/*pirB*-positive and *pirA*/*pirB*-negative groups were performed using Python (v3.13) with the SciPy library. Differences between groups were evaluated using the non-parametric Mann–Whitney *U* test.

## 3 Results and discussion

### 3.1 Whole-genome characterization of *Vibrio campbellii* HJ-2023 and *Vibrio campbellii HJ*-2023n

Whole-genome sequencing and structural analysis were conducted on two *V. campbellii* strains isolated from Taean-gun, Chungcheongnam-do, Republic of Korea: the pathogenic AHPND strain *V. campbellii* HJ-2023 and the non-pathogenic strain *V. campbellii* HJ-2023n.

**Figure 1:**
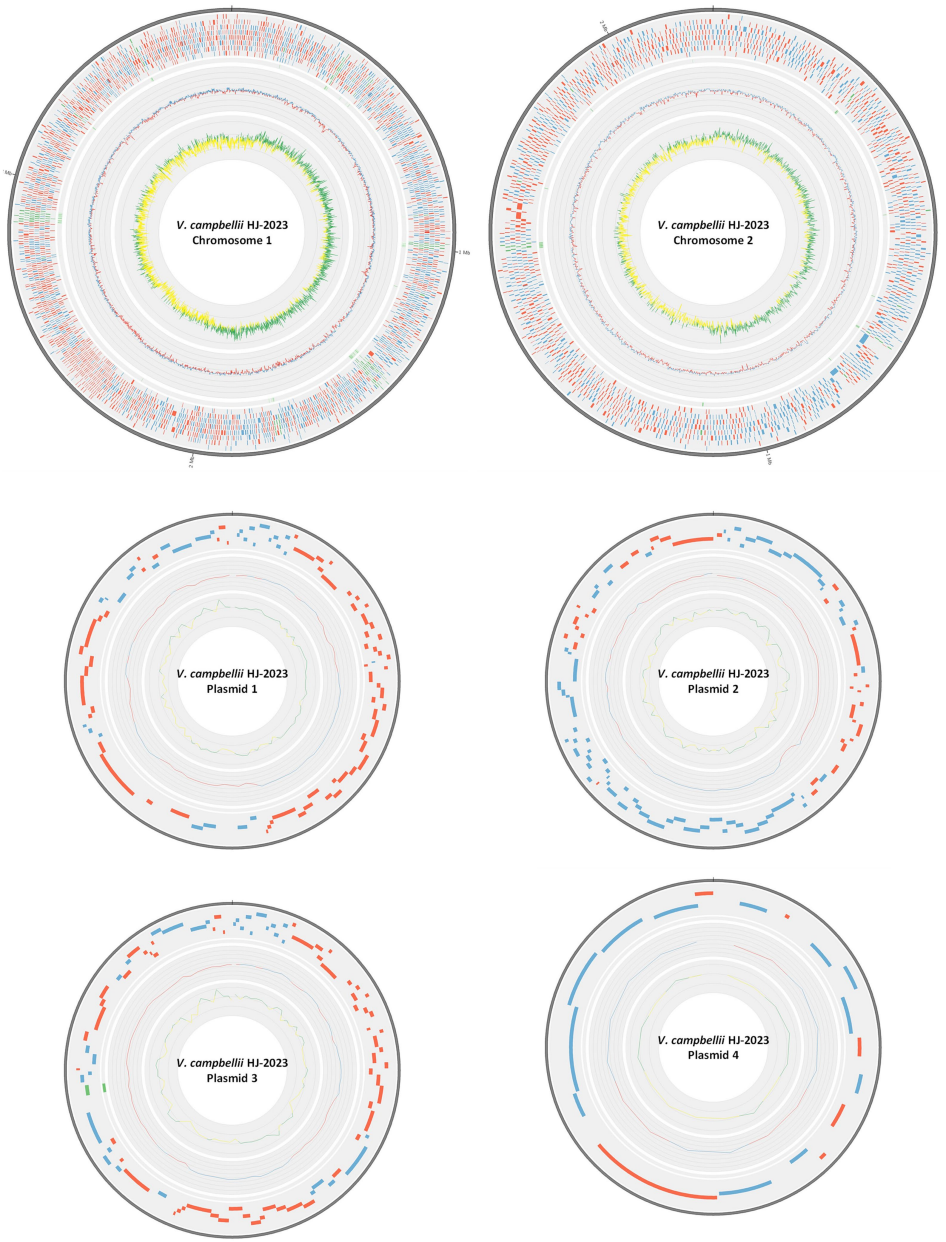
Genomic structure of *Vibrio campbellii* strain HJ-2023. The circular maps (from outermost to innermost) represent two chromosomes (Chromosome 1 and Chromosome 2) and four plasmids (Plasmids 1–4). Each map includes concentric rings showing: predicted coding sequences on the forward strand (blue) and reverse strand (red), with virulence-related genes highlighted in green; GC content (blue bars, ≥average; red bars, <average); and GC skew (green, ≥average; yellow, <average).

The complete genome of *V. campbellii* HJ-2023 comprised two chromosomes and four plasmids (Figure 1 and Table 1), whereas *V. campbellii* HJ-2023n assembled into two chromosomes and a single plasmid (Supplementary Figure S1 and Supplementary Table S1).

**Table 1:** Genome statistics of *Vibrio campbellii* HJ-2023.

Genomic feature	Total value	Chromosome 1	Chromosome 2	Plasmid 1	Plasmid 2	Plasmid 3	Plasmid 4
Size of the genome assembly (bp)	6,236,048	3,790,591	2,167,482	88,782	87,228	86,174	15,791
GC content (%)	45.40	45.43	45.34	45.83	45.71	45.41	43.91
Protein-coding genes	5,531	3,324	1913	90	98	89	17
tRNA/tmRNA/rRNA genes	137/1/37	122/1/34	15/0/3	—	—	—	—
Complete BUSCOs (%)	98.39						

Whole-genome alignment revealed extensive collinearity between the two isolates at the chromosomal level, confirming that their overall genomic architecture is highly conserved despite differences in plasmid content (Supplementary Figure S2).

Both strains were initially identified as *V. campbellii* based on 16S rRNA sequence analysis (Choi et al., 2024). However, species-level classification in bacteria often requires genomic analyses beyond 16S rRNA genes (Saygin et al., 2020; Antony-Babu et al., 2017; Maiti and Mandal, 2021; Kusuma et al., 2020). To verify pairwise genome sequence identity, OrthoANI was calculated through genome-based comparisons with *Vibrio* type strains. Although all comparisons with other *Vibrio* species produced ANI values below the 95% threshold for species delineation (Cui et al., 2019), both isolates showed the highest nucleotide identity with the *V. campbellii* reference strain (*V. campbellii* BoB-53; GCF_002906475.1). And for the precise definitions of taxonomic, dDDH similarity values were calculated using the GGDC web server. The dDDH values between HJ-2023 and *V. campbellii* type strain and HJ-2023n and *V. campbellii* type strain (*V. campbellii* 200612B; GCF_000400205.1, *V. campbellii* 051011F; GCF_000818315.1) were 80.1 and 79.6%, respectively, both greater than the threshold value of 70% for determination as a species (Supplementary Figures S3, S4). Accordingly, based on OrthoANI and dDDH metrics, both isolates were confidently classified as *V. campbellii* (Supplementary Tables S2–S5).

### 3.2 Virulence factor-based differentiation of AHPND and non-AHPND *Vibrio campbellii* strains

**Figure 2:**
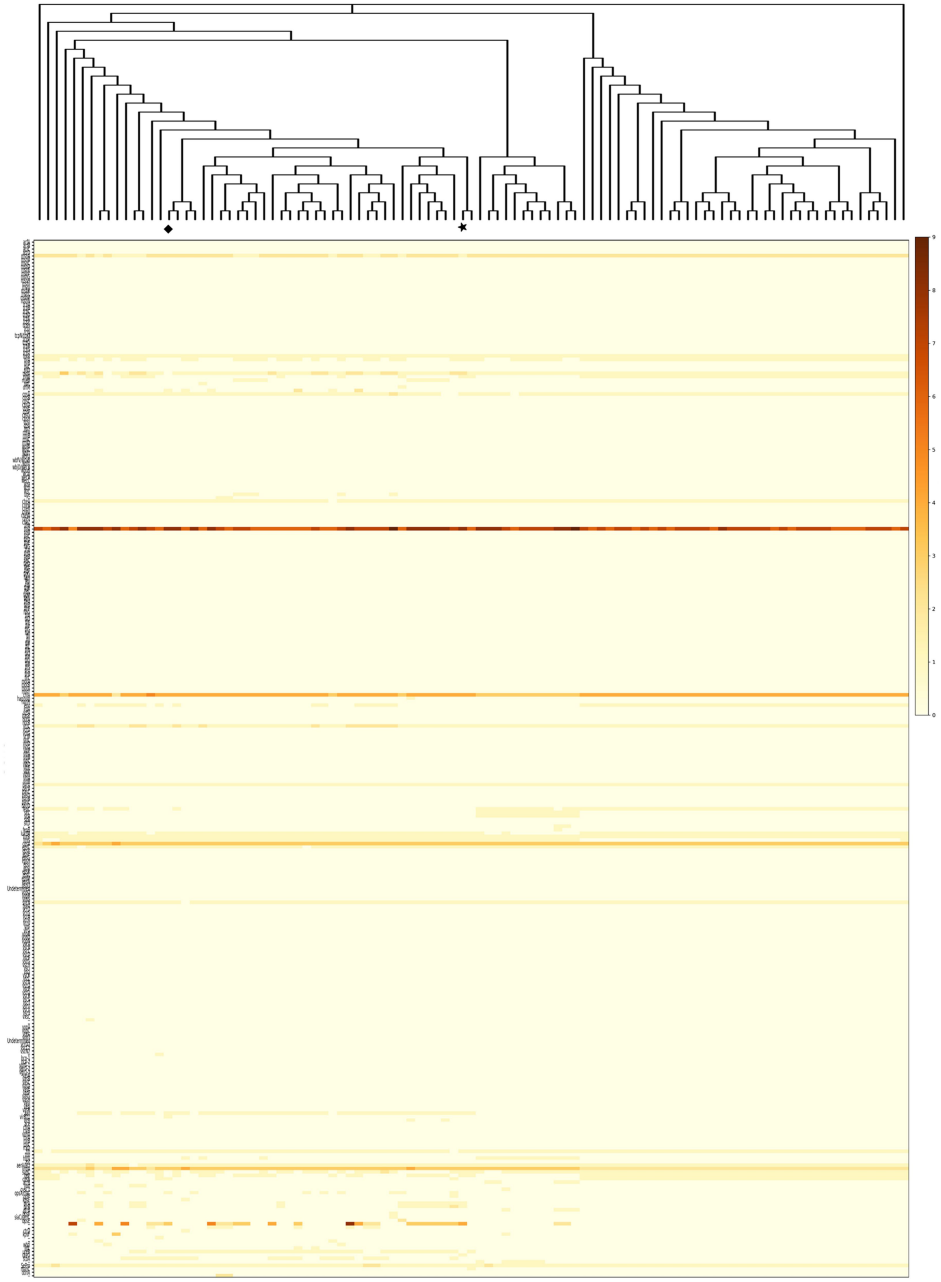
Phylogenetic relationships and gene presence matrix among *Vibrio campbellii* strains. The top dendrogram shows a phylogenetic tree constructed from whole-genome sequence alignments. The heatmap on the right depicts the presence and relative similarity of selected virulence factor genes (*x*-axis) across strains (*y*-axis). Gene presence is indicated by color intensity, with the accompanying scale bar representing similarity scores (red = high, yellow = moderate, white = absent/low). A vertical annotation bar between the tree and heatmap indicates strain groupings: *pirA/pirB*-positive strains (including *V. campbellii* HJ-2023, marked with a star) are shown in red, and *pirA/pirB*-negative strains (including *V. campbellii* HJ-2023n, marked with a diamond) in blue.

Virulence factor profiling was conducted across 101 *V. campbellii* strains, including the two isolates from this study. Among them, 12 strains—including the pathogenic *V. campbellii* HJ-2023—harbored the *pirA* and *pirB* genes on a plasmid, which are established pathogenic determinants of AHPND (Lightner et al., 2015). Conversely, the remaining 89 non-pathogenic strains lacked these toxin genes (Figure 2).

Comprehensive characterization of the 89 *pirA/pirB*-negative strains identified 24 virulence factor genes (Supplementary Table S6). Most, including motility genes (*cheA* and *flaA*) (Echazarreta et al., 2018; Xu et al., 2021) and iron acquisition genes (*barA*) (Zhang et al., 2020), were universally distributed across all genomes and thus are unlikely to serve as distinctive markers for this group. By contrast, adhesion-associated genes such as *mshA*, *tadA*, and *toxR* (Taiwo et al., 2017; Jones et al., 2015) were relatively enriched, suggesting potential roles in biofilm formation and environmental persistence. These factors likely contribute to survival in marine niches rather than direct pathogenicity.

By contrast, the 12 *pirA/pirB*-positive strains, including *V. campbellii* HJ-2023, carried not only the AHPND toxin genes but also additional virulence-associated genes, such as those involved in host invasion (*pilA*) (Paranjpye and Strom, 2005), metabolic efficiency (*sugC*) (Hu et al., 2024), immune evasion (*lgtF*) (Hu et al., 2024), and toxin regulation and secretion (*sycN*) (Iriarte and Cornelis, 1999). Although these genes were not exclusive to this subgroup, their presence may enhance adhesion, metabolic adaptation, and immune evasion. Moreover, enzymatic toxins, including hemolysins and zinc metalloproteases (Benitez and Silva, 2016), were identified, potentially increasing tissue invasion capacity.

Overall, although no virulence gene was exclusively restricted to the 12 *pirA/pirB*-positive strains, this subgroup showed a tendency to harbor additional virulence determinants. Such enrichment may partly account for the enhanced pathogenic potential observed in infection models, although further functional validation is necessary.

### 3.3 Pan-genomes analysis of *Vibrio campbellii* strains

**Figure 3:**
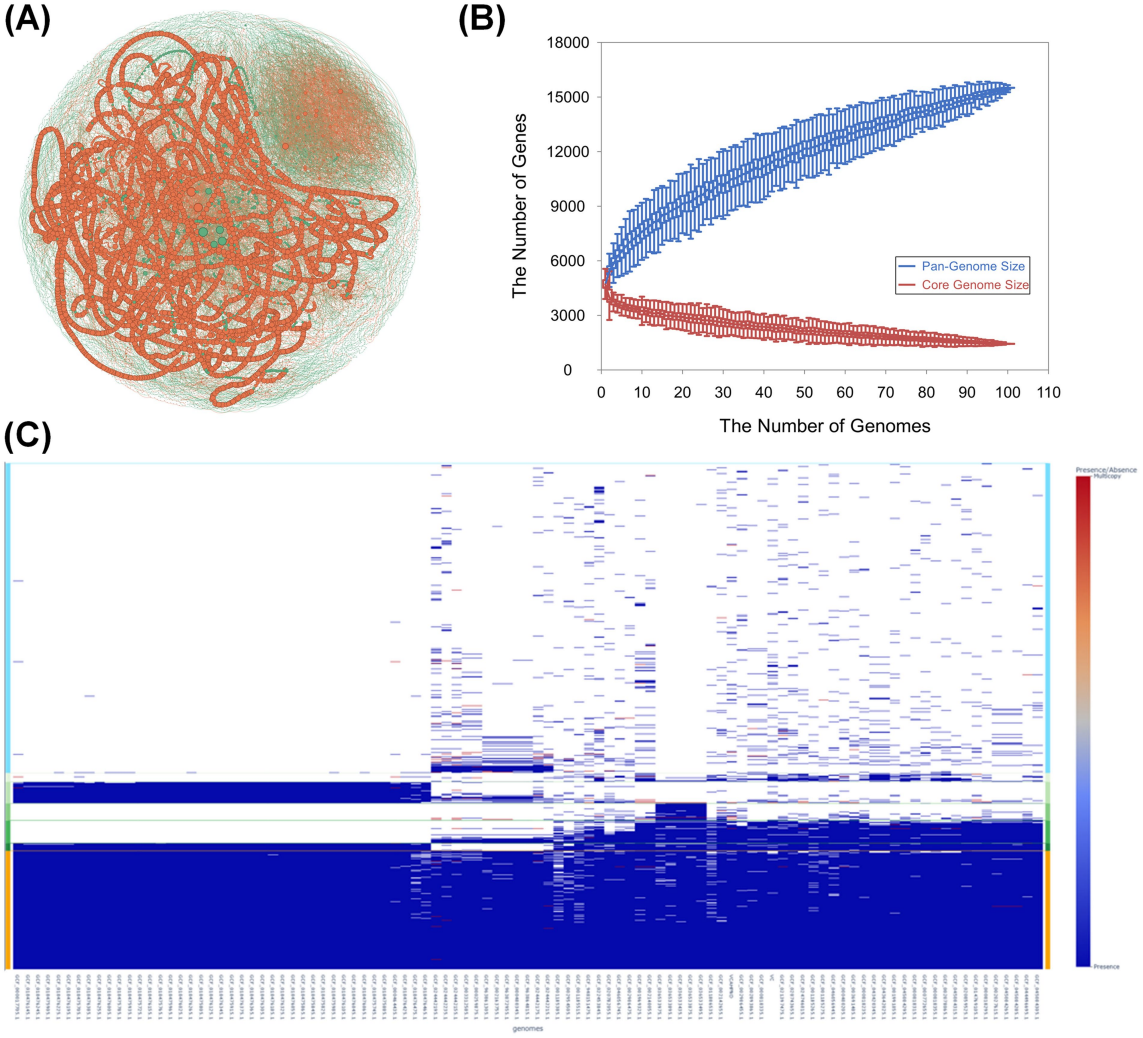
Comparative genomic analysis of *Vibrio campbellii* strains. **(A)** Gene co-occurrence network of 101 genomes constructed with Gephi (ForceAtlas2 layout). Each node represents a gene cluster, with edges connecting clusters co-occurring in the same genomes. Node size reflects degree centrality, and node color indicates genome partition (orange = *pirA/pirB*-positive strains; green = *pirA/pirB*-negative strains). Although the two groups do not show a clear visual separation due to network complexity, pathogenic modules are enriched in transposase families (IS5, IS630, and IS30-like). **(B)** Rarefaction curves illustrating pangenome expansion with additional genomes. The *x*-axis shows the number of genomes analyzed, and the *y*-axis shows the number of gene clusters. Curves represent the total pangenome (black), core genome (magenta), soft-core (brown), shell (cyan), and cloud genes (red/orange), consistent with an open pangenome structure. **(C)** Gene presence/absence matrix across all genomes. Genes are shown on the *x*-axis and genomes on the *y*-axis. Blue indicates gene presence and white indicates absence. A vertical color bar to the right summarizes overall identity or abundance scores, highlighting distribution differences between *pirA/pirB*-positive and *pirA/pirB*-negative strains.

A pan-genome analysis of 101 *V. campbellii* genomes identified 15,482 gene families. Among these, 3,624 (23.40%) belonged to the Persistent group, 2,386 (15.38%) to the Shell group, and 9,472 (61.22%) to the Cloud group (Figure 3). Consistent with the previously reported high genomic variability and active HGT within the genus *Vibrio* (Deng et al., 2019), this distribution underscores an open pan-genome structure in *V. campbellii*, reflecting the continual acquisition of new gene families.

Using gene presence/absence data, network analysis revealed a tendency toward separation between *pirA/pirB*-positive strains (12 genomes, including AHPND *V. campbellii* HJ-2023) and *pirA/pirB*-negative strains (89 genomes, including *V. campbellii* HJ-2023n). Although this separation is not visually distinct in Figure 3A due to network complexity, the modular organization highlights the role of mobile genetic elements (Xiao et al., 2017). In particular, transposase genes from the IS5, IS630, and IS30-like families frequently clustered within pathogenic groups, consistent with their known involvement in carrying virulence determinants, maintaining genomic stability, and facilitating horizontal transfer. Moreover, in *pirA/pirB*-positive strains, several hub genes with high network centrality (degree >150) were identified, including the IS5 family transposase (*HAS37_RS23470*), the IS630 family transposase (*HAS37_RS07130*), and the IS30-like element ISYm1 transposase (*LL295_RS20420*). These findings strongly suggest that the intergenomic transfer and stabilization of *pirA/pirB* toxin genes between plasmids and chromosomes are likely facilitated by transposase-mediated mobility.

In addition to modules enriched with transposase genes, identified through functional annotation and supported by gene presence/absence data (Supplementary Table S7), the network also contained clusters of membrane-associated proteins, toxin-antitoxin systems, and various transferases (Figure 3A). Although not unique to *pirA/pirB*-positive strains, these clusters may provide secondary adaptive traits, such as enhanced host interaction or stress tolerance. The observed modular organization indicates that the pathogenic potential of *V. campbellii* is not solely driven by *pirA/pirB* toxin genes but is also influenced by interactions among accessory genomic elements.

The *pirA/pirB*-negative group exhibited two distinct trends. First, strain-specific genes were frequently located at the periphery of the Cloud gene group, suggesting a role in environmental adaptability. This pattern may also reflect evolutionary processes such as niche specialization or divergence from a shared ancestral gene pool. Second, compared with the *pirA/pirB*-positive group, the negative group lacked several high-centrality hubs in the co-occurrence network; in the positive group, prominent hubs included transposase genes such as the IS4 family transposase (*HAS35_RS143*) and the IS5 family transposase (*HAS37_RS22*) (Supplementary Table S7). Collectively, these contrasts suggest that transposase-mediated genome dynamics contribute to maintaining genomic variability, independent of *pirA/pirB*-dependent pathogenicity.

t-SNE analysis based on the pan-genome presence/absence matrix revealed distinct clustering within the *pirA/pirB*-negative group, whereas the *pirA/pirB*-positive strains were scattered across multiple regions (Supplementary Figure S5). This suggests that *pirA/pirB*-negative strains exhibit greater genomic homogeneity. Notably, the presence of *pirA* and *pirB* toxin genes across diverse genomic backgrounds underscores their acquisition through HGT.

In addition, two genes—*AAFX21_RS08865*, encoding a hypothetical protein of unknown function, and *AAFX21_RS08870*, encoding an AAA family ATPase—were consistently detected in *pirA/pirB*-positive strains. Considering the established role of AAA family ATPases in cellular homeostasis, stress response, and the regulation of pathogenic processes, these genes represent promising candidates for further investigation into the molecular determinants of *V. campbellii* pathogenicity.

### 3.4 Distribution and functional implications of insertion sequence elements

Mobile genetic elements, such as IS elements, are essential for genomic rearrangements, including HGT, and for regulating virulence gene expression in bacteria (Johansson et al., 2025). However, a genome-wide survey of IS elements across all 101 *V. campbellii* strains revealed no statistically significant differences in either the number of IS elements or the diversity of IS families between the *pirA/pirB*-positive and *pirA/pirB*-negative groups.

**Figure 4:**
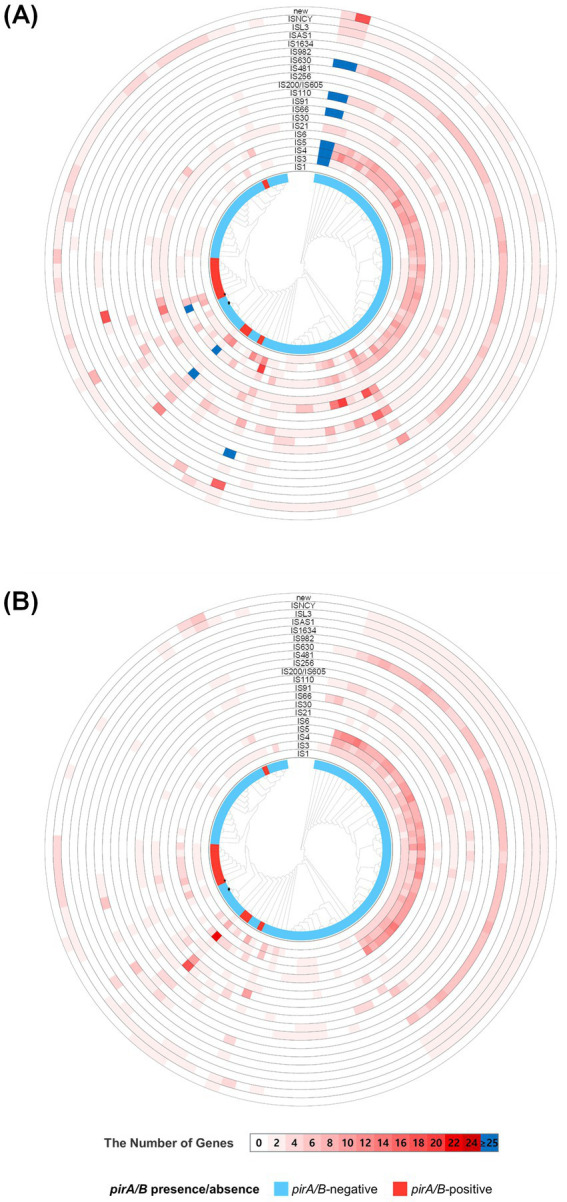
Distribution of insertion sequence (IS) elements among *Vibrio campbellii* strains. **(A)** Circular plot showing the presence and abundance of IS element families across the genomes. The central phylogenetic tree is based on whole-genome similarity. Concentric rings represent different IS families (e.g., IS1, IS3, ISNCY, ISL3, IS982, ISAS1, IS630, IS200/IS605, IS256, IS110, IS1634, IS481, IS91, IS30, IS21, IS5, IS4, IS66, IS6, and novel IS types). Each position on a ring corresponds to the copy number of that IS element in a given strain. **(B)** Color scale for IS gene counts (0 to ≥25) and strain annotation: light blue for *pirA/pirB*-negative strains and red for *pirA/pirB*-positive strains. *V. campbellii* HJ-2023 is marked with a star (★), and HJ-2023n with a diamond (◆).

Genome analysis revealed that IS families such as IS3, IS5, IS30, IS630, and ISNCY were more frequently detected than others (Supplementary Table S8). Although certain pathogenic strains exhibited a higher frequency of specific IS families (Figure 4), no significant differences were observed in the total load of chromosomal IS elements between the *pirA*/*pirB*-positive and *pirA*/*pirB*-negative groups (Mann–Whitney *U* test, *p* = 0.23; Supplementary Table S12 and Supplementary Figure S6). Comparative analysis between *pirA*/*pirB*-positive and *pirA*/*pirB*-negative strains indicated that the presence of mobile genetic elements, including IS elements and transposases, did not significantly differ. This indicates that the acquisition of the AHPND plasmid did not result in extensive genomic rearrangements in the *V. campbellii* isolate. This is in contrast to some very clonal outbreaks of *V. parahaemolyticus*, in which large increases in IS elements frequently coincide with the acquisition of a virulence plasmid (Xiao et al., 2017). Instead, our results suggest that the genomic background of *V. campbellii* is relatively relatively unchanged, and that its transition to a pathogenic genotype appears to be primarily associated with a gene-acquisition event involving pirA and pirB, which likely acts as a key determinant. These findings highlight the importance of strain-level structural genomic studies, as the average IS content alone does not sufficiently explain virulence potential. Consistent with the pan-genome analysis, the 12 *pirA/pirB*-positive strains, including *V. campbellii* HJ-2023, carried *pirA* and *pirB* toxin genes in close proximity to IS elements, such as the IS5 family transposase (*HAS37_RS23470*), the IS630 family transposase (*HAS37_RS07130*), and the IS30-like transposase (*LL295_RS204*) (Deng et al., 2019). Conversely, although similar IS families were also present in the non-pathogenic *V. campbellii* HJ-2023n strain, no co-localization with virulence genes was detected. This comparison suggests that the genomic context of IS elements—including their proximity to virulence-associated regions—may play a role in the acquisition or regulation of pathogenic traits beyond their simple presence. Supporting this interpretation, a Tn3-like transposon has previously been reported to insert into the AHPND-associated pVA1 plasmid in *V. parahaemolyticus* (Kumar et al., 2021).

Therefore, although group-level comparisons did not reveal a direct association between IS element abundance and pathogenicity, the comparative analysis of the two focal strains indicates that the structural positioning and clustering of IS elements with virulence genes may be associated with pathogenicity-related genomic contexts (Siguier et al., 2014). These findings underscore the importance of an integrated analytical framework for virulence prediction—one that accounts not only for the presence of virulence genes but also for their broader genomic context, particularly the structural associations with IS elements.

### 3.5 Comparative analysis of secretion system gene clusters

**Figure 5:**
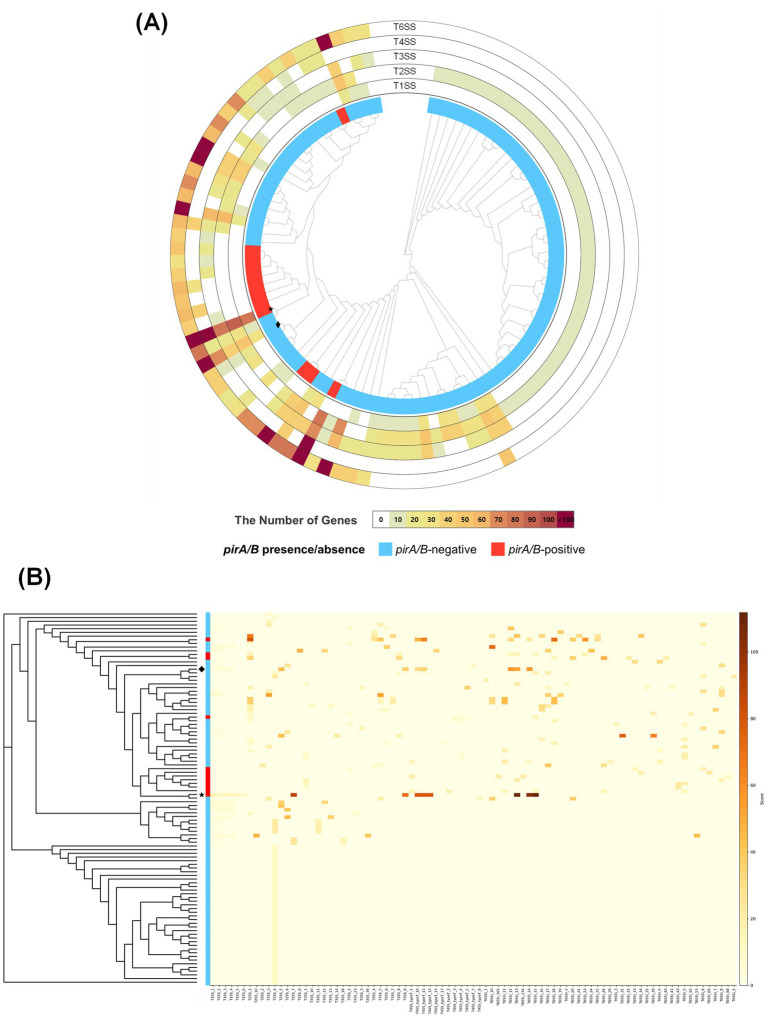
Distribution of secretion system-associated genes among *Vibrio campbellii* strains. **(A)** Circular plot showing the number and distribution of genes for various bacterial secretion systems across genomes. The central phylogenetic tree represents whole-genome relationships. Concentric rings correspond to *T1SS*, *T2SS*, *T3SS*, *T4SS*, and *T6SS* (innermost to outermost), with color intensity reflecting gene counts (light yellow = low, dark brown = high). **(B)** Heatmap of secretion system gene distribution across strains (*x*-axis: gene clusters; *y*-axis: strains). The dendrogram on the left shows phylogenetic relationships. Strains are annotated by *pirA/pirB* status: *pirA/pirB*-positive strains in red, *pirA/pirB*-negative strains in light blue. *V. campbellii* HJ-2023 is marked with a star (★), and HJ-2023n with a diamond (◆).

Different protein secretion systems facilitate the delivery of toxic proteins, enzymes, and exotoxins into host cells, thereby enhancing bacterial pathogenicity. In this study, the distribution of secretion system gene clusters was analyzed across 101 *V. campbellii* genomes using the DeepSecE tool. Most strains carried genes associated with Type I (*T1SS*) and Type II (*T2SS*) secretion systems. In addition, several pathogenic strains harbored gene clusters corresponding to Type III (*T3SS*) or Type VI (*T6SS*) secretion systems (Figure 5).

Despite these findings, neither the total number nor the overall composition of secretion system genes differed significantly between pathogenic and non-pathogenic groups. This suggests that the mere presence of secretion system genes is insufficient to predict pathogenic potential.

Closer examination of the two Korean isolates, *V. campbellii* HJ-2023 and *V. campbellii* HJ-2023n, revealed marked differences in the quantity and completeness of *T1SS* and *T6SS* gene clusters. *V. campbellii* HJ-2023 contained a higher number of these genes and more complete cluster configurations. Notably, the *T6SS* gene cluster in *V. campbellii* HJ-2023 included multiple copies of key structural components, such as Hcp (hemolysin-coregulated protein), VgrG (valine-glycine repeat protein G), TssB, and TssC, which together form the injection apparatus comprising the tube, spike, and contractile sheath (Supplementary Table S9).

Conversely, *V. campbellii* HJ-2023n carried only partial sets of *T6SS* components or lacked several altogether, suggesting that the *T6SS* in this strain may be incomplete and thus potentially less functional. In addition, *V. campbellii* HJ-2023 possessed duplicated gene clusters for the *T1SS*, including membrane fusion proteins and ATP-binding cassette transporters. This arrangement may provide a more robust genetic basis to support secretion function.

These findings indicate that *V. campbellii* HJ-2023 harbors a more complete repertoire of *T1SS* and *T6SS* genes, which may contribute to its virulence potential beyond the effects of the *pirA* and *pirB* toxin genes alone. A notable strain-level difference between the two focal isolates may therefore lie in this expanded repertoire of secretion-related genes. In particular, *T6SS* systems may act directly on host cells or competing bacteria, thereby broadening the spectrum of virulence in ways that may extend beyond the action of *pirA* and *pirB*. A statistical analysis indicated that the combined abundance of secretion system genes was significantly greater in the *pirA*/*pirB*-positive group (Mann–Whitney *U* test, *p* = 0.005), which was mainly accounted for by enrichment of T6SS clusters (*p* < 0.001; Supplementary Table S12 and Supplementary Figure S7).

### 3.6 Analysis of antibiotic resistance genes in *Vibrio campbellii* strains

Most of the 101 *V. campbellii* strains analyzed in this study carried at least one antibiotic resistance gene, as identified through the CARD and the NCBI antibiotic resistance gene databases. Previous studies have shown that plasmid- and IS element-associated resistance genes are commonly present in AHPND-causing *V. parahaemolyticus* and other *Vibrio* species (Kitiyodom et al., 2010).

**Figure 6:**
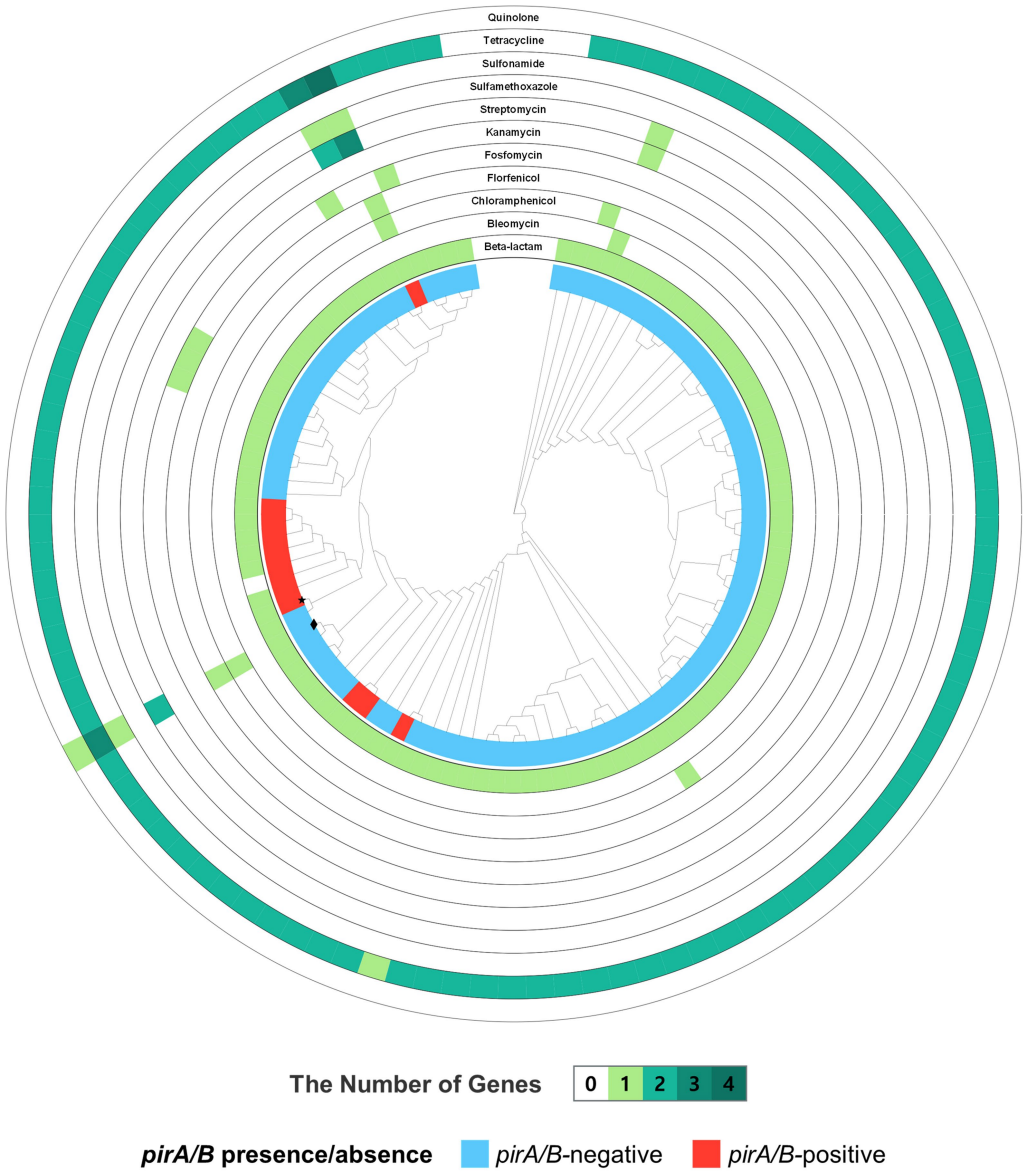
Distribution of antibiotic resistance genes among *Vibrio campbellii* strains. Circular phylogenetic diagram based on whole-genome similarity (center tree). Concentric rings indicate the presence (light green) and abundance (darker green) of individual antibiotic resistance genes, with gene names labeled on the outermost track. Genes include determinants for aminoglycoside, tetracycline, sulfonamide, *β*-lactam, chloramphenicol, fluoroquinolone, and fosfomycin resistance (e.g., *aph(3″)-Ib*, *tetR*, *tet(35)*, *tetA(D)*, *sul2*, *fosA*, *catA2*, *floR*, *blaVHW-1*, and *qnrS2*). The color scale at the bottom represents gene counts (0 = white to 2 = dark green). Strains are annotated by AHPND status: AHPND-positive in red, non-AHPND in light blue. *V. campbellii* HJ-2023 is marked with a star (★), and HJ-2023n with a diamond (◆).

No consistent or notable differences in antibiotic resistance gene content were observed between pathogenic (AHPND) and non-pathogenic *V. campbellii* groups at the group level (Figure 6). Specifically, statistical analysis confirmed that there was no significant difference in the total count or richness of antibiotic resistance genes between the *pirA*/*pirB*-positive and *pirA*/*pirB*-negative groups (Mann–Whitney *U* test, *p* = 0.43; Supplementary Table S12 and Supplementary Figure S8). However, certain pathogenic strains carried more resistance genes, suggesting that resistance gene abundance may be more strongly influenced by strain-specific genomic architecture or selective environmental pressures than by pathogenicity itself.

Direct comparisons between the two strains isolated in this study, *V. campbellii* HJ-2023 and *V. campbellii* HJ-2023n, revealed clear variations. The pathogenic AHPND strain *V. campbellii* HJ-2023, with sequence homologies of 83.44, 98.65, and 99.06%, carried a limited set of antibiotic resistance genes, including *tet(34)*, *tet(35)*, and *blaVHH-1* (Supplementary Table S10).

By contrast, the non-pathogenic strain carried additional resistance genes, primarily those conferring resistance to quinolones, sulfonamides, and streptomycin. This suggests that non-pathogenic strains may harbor more complex resistance gene profiles, as the relationship between pathogenicity and antibiotic resistance at the genomic level appears inconsistent. These findings further support the notion that antibiotic susceptibility is likely influenced by gene expression levels and functional activity, consistent with the susceptibility tests of *V. campbellii* HJ-2023, which demonstrated sensitivity to tetracycline and oxytetracycline. Phylogenetic visualization (Figure 6) highlights these variations and clearly differentiates the two strains based on their resistance gene profiles.

These findings suggest that pathogenic *Vibrio* strains do not inherently possess elevated antibiotic resistance, underscoring the importance of precise strain-level investigations to assess accurately resistance risks. Accordingly, the relatively antibiotic-susceptible profile of *V. campbellii* HJ-2023 provides valuable baseline information for tracking the emergence of resistance and guiding targeted antibiotic therapies.

### 3.7 CAZyme profile and its role in environmental adaptation and virulence

Comparative genomic analysis of CAZymes across 101 *V. campbellii* strains revealed complex distribution patterns among CAZyme (sub)families. Several subfamilies were consistently conserved across most genomes, indicating that carbohydrate utilization capacity is broadly maintained regardless of pathogenicity status, likely reflecting a common ecological strategy within the *Vibrio* genus.

**Figure 7:**
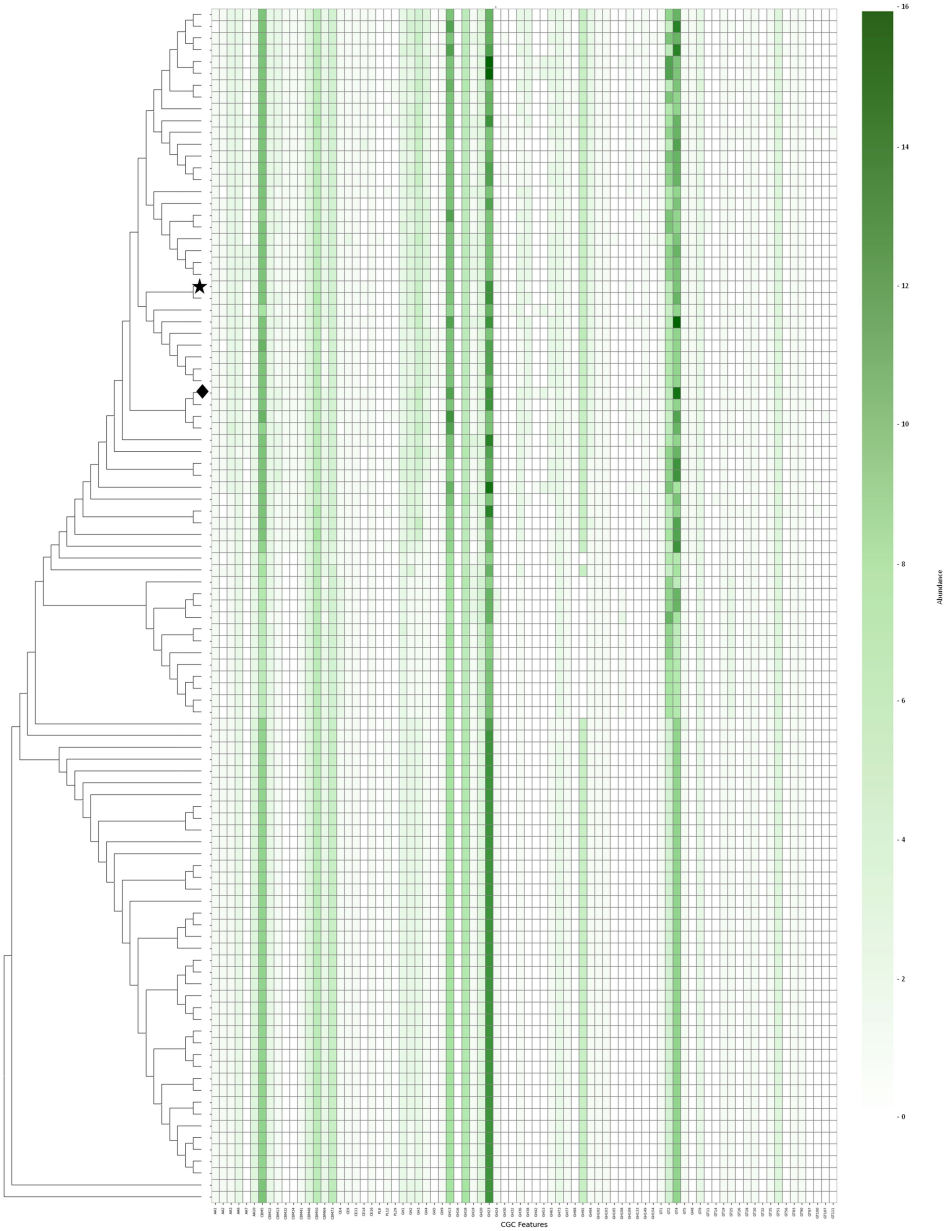
Distribution of carbohydrate gene clusters (CGCs) across *Vibrio campbellii* strains. Heatmap showing the presence and abundance of CGCs. Each row represents a genome, and each column represents a distinct CGC. Color intensity reflects gene count per CGC type, with darker green indicating higher abundance. Dendrogram on the left depicts genome clustering based on CGC profiles.

All strains carried diverse glycoside hydrolase (GH) and glycosyltransferase (GT) enzymes, notably the chitin-degrading enzymes GH18 and GH20 (Li and Roseman, 2004) (Figure 7). These enzymes are often associated with chitin-binding modules, which enhance attachment to insoluble substrates and thereby facilitate efficient degradation (Perrakis et al., 1994). The widespread distribution of these genes allows *V. campbellii* and other marine *Vibrio* species to play a key role in carbon and nitrogen cycling in chitin-rich aquatic environments (Meibom et al., 2005). Furthermore, the consistent presence of chitinolytic enzymes in both pathogenic and non-pathogenic strains highlights a common ecological adaptation to shrimp exoskeletons and related environmental niches.

Statistical analysis also showed that *pirA*/*pirB*-positive strains carried a significantly higher number of total CAZyme genes (Mann–Whitney *U* test, *p* = 0.008) and Glycoside Hydrolases (*p* = 0.003) than non-pathogenic strains (Supplementary Table S12 and Supplementary Figure S9). Although not known to be toxic, the enzymes reported in this study (Supplementary Table S7) may indirectly contribute to virulence by promoting host colonization. During the early stages of infection, chitin-rich barriers—such as those targeted by chitinase-producing bacteria—have been associated with host invasion via *ChiA* and *ChiD* (Li and Roseman, 2004; Meibom et al., 2005). Consistent with this, a 2024 study by Deng et al. (2025) demonstrated that *Vibrio* pathogens exhibit active chitin-degrading activity on shrimp shell substrates, accompanied by a marked increase in chitinase gene expression.

Interestingly, machine learning modeling of CAZyme gene distributions indicated that CAZyme profiles could help distinguish genomes carrying *pirA/pirB* genes from those lacking them. Specifically, the Gradient Boosting model achieved the highest predictive performance (AUC = 0.881, F1 = 0.905), demonstrating promising classification performance within the current dataset (Supplementary Figure S10 and Supplementary Table S13). Among the most informative features, GH63 was frequently ranked as a key marker. Although this does not confirm a direct functional role in pathogenicity, it suggests that specific CAZyme families may serve as genomic indicators for strain classification.

Although not essential for pathogenicity, these additional enzymes may enhance colonization in the primary host and increase the potency of other virulence factors. Moreover, CAZyme gene patterns did not directly correlate with pathogenic potential in *V. campbellii* strains, underscoring the likely critical role of specific virulence determinants such as *T6SS* clusters and *pirA* and *pirB* toxins. Nevertheless, CAZymes may act as accessory factors by promoting adherence to host chitin structures and facilitating synergistic interactions with primary virulence mechanisms.

Finally, while this study focused on genomic elements directly associated with HGT and host interaction, future investigations should extend to regulatory networks, such as Quorum Sensing and CRISPR/Cas systems, to elucidate the precise regulatory mechanisms governing virulence gene expression in *V. campbellii*.

## 4 Conclusion

This study identified genomic features associated with AHPND-linked signatures through a comparative genomic analysis of 101 *V. campbellii* strains. Although the *pirA* and *pirB* toxin genes were confirmed as essential, additional genomic features—including gene clustering, the positioning of transposable elements, and the expansion of secretion systems—were also identified as putative genomic factors that may be associated with increased virulence potential.

Notably, the recently isolated *V. campbellii* HJ-2023 strain from the Republic of Korea exhibited distinct structural features, including expanded *T6SS* and *T1SS* gene clusters and key mobile genetic elements flanking the toxin genes. These characteristics highlight this strain as a valuable reference genome for monitoring regional emergence and guiding future risk assessments.

Although CAZyme profiles and antibiotic resistance showed no clear association with pathogenicity, certain enzymes, such as chitinases, may facilitate infection spread. Overall, this study underscores that AHPND pathogenicity likely involves multifactorial genetic interactions and provides critical insights to support improved management and diagnostic strategies in shrimp aquaculture.

## Data Availability

The complete genome sequences of the pathogenic and non-pathogenic *Vibrio campbellii* strains were deposited in NCBI GenBank under accession numbers JBRBAD000000000 and JBRBAE000000000 (BioProject: PRJNA1321280).

## Footnotes

1
http://www.mgc.ac.cn/VFs/
