# Supplementary material for: Genome-based characterization of AHPND and non-AHPND Vibrio campbellii isolates from Republic of Korea
Source: Front Microbiol. 2026 Jan 26;17:1724818. doi: 10.3389/fmicb.2026.1724818 (PMC12883650; doi:10.3389/fmicb.2026.1724818)
Supplement: Supplementary file 1 [file Data_Sheet_1.docx]

**Supplementary data**

**Supplementary Figure S1. Genomic structure of the *Vibrio* *campbellii* strain HJ-2023.** From the outermost to the innermost circles, the circular maps display two chromosomes (Chromosome 1 and Chromosome 2) and single plasmid (Plasmid 1). Each map consists of multiple concentric rings: predicted coding sequences on the forward strand (blue) and reverse strand (red), with predicted virulence-related genes highlighted in green; GC content (blue bars indicate regions with GC content ≥ average, red bars indicate < average); and GC skew (green for GC skew ≥ average, yellow for < average).

**Supplementary Figure S2. Alignment between chromosomal sequences of the two strains.** Ribbons with blue color indicate the same strand and the red for inverted strands. Hits retrieved from megablast were shown in the figure.

**Supplementary Figure S3. Distribution of genomic relatedness for strain HJ-2023.** The scatter plot illustrates the distribution of OrthoANI and dDDH values calculated between strain HJ-2023 and 258 selected *Vibrio* genomes. The X- and Y-axes represent the calculations of OrthoANI and dDDH against the genome sequence of strain HJ-2023, respectively.

**Supplementary Figure S4. Distribution of genomic relatedness for strain HJ-2023n.** The scatter plot illustrates the distribution of OrthoANI and dDDH values calculated between strain HJ-2023n and 258 selected *Vibrio* genomes. The X- and Y-axes represent the calculations of OrthoANI and dDDH against the genome sequence of strain HJ-2023n, respectively.

**Supplementary Figure S5. t-SNE visualization of genomic similarity among *Vibrio campbellii* strains*.*** t-SNE scatter plot showing clustering of strains based on genomic features. Strains are colored by *pirA*/*pirB* present status: red dots indicate *pirA*/*pirB*-negative strains and blue dots indicate *pirA*/*pirB*-positive strains.

**Supplementary Figure S6. Boxplots comparing insertion sequence (IS) abundance and richness between *pirA*/*pirB*− and *pirA*/*pirB*+ genomes.** (A) total IS counts in chromosomes, (B) total IS counts in plasmids, (C) IS family richness in chromosomes, and (D) IS family richness in plasmids. Individual points represent genomes. Statistical differences were assessed using the Mann–Whitney U test.

**Supplementary Figure S7. Boxplots comparing secretion system (SS) hits between pirA/B− and pirA/B+ genomes.** (A) total SS hits, (B) type I secretion system (T1SS) hits, (C) type II secretion system (T2SS) hits, (D) type III secretion system (T3SS) hits, (E) type IV secretion system (T4SS) hits, and (F) type VI secretion system (T6SS) hits. Individual points represent genomes. Statistical differences were assessed using the Mann–Whitney U test. (G) Bar plot showing the presence rate of each secretion system (any hit) in *pirA*/*pirB*− and *pirA*/*pirB*+ genomes.

**Supplementary Figure S8. Boxplots comparing antibiotic resistance gene (ARG) profiles between *pirA*/*pirB-* and *pirA*/*pirB*+ genomes.** (A) total ARG hits, (B) ARG gene richness, and (C) ARG class richness. Individual points represent genomes. Statistical differences were assessed using the Mann–Whitney U test.

**Supplementary Figure S9. Boxplots comparing total CAZyme counts and major CAZyme classes between *pirA*/*pirB*− and *pirA*/*pirB*+ genomes.** (A) CAZyme total, (B) auxiliary activities (AA), (C) carbohydrate-binding modules (CBM), (D) carbohydrate esterases (CE), (E) glycoside hydrolases (GH), and (F) glycosyltransferases (GT). Individual points represent genomes. Statistical differences were assessed using the Mann–Whitney U test.

**Supplementary Figure S10. Top five importance features based on decrease in area under the curve (AUC) for four machine learning models applied to classification on *pirA/B* presence and absence.** (A) Gradient Boosting, (B) Logistic Regression, (C) Random Forest, and (D) Naive Bayes models showing the relative contribution of genomic features to model performance. Features are ranked by the magnitude of AUC decrease when removed from the model, with larger decreases indicating greater importance for model performance. Error bars represent standard deviation across cross-validation folds.

**Fig. S1**

**Fig. S2**

**Fig. S3**

**Fig. S4**

**Fig. S5**

**Fig. S6.**

**Fig. S7**

**Fig. S8**

**Fig. S9**

**Fig. S10**
