# Supplementary material for: Genome-based characterization of AHPND and non-AHPND Vibrio campbellii isolates from Republic of Korea
Source: Front Microbiol. 2026 Jan 26;17:1724818. doi: 10.3389/fmicb.2026.1724818 (PMC12883650; doi:10.3389/fmicb.2026.1724818)
Supplement: Supplementary file 2 [file Supplementary_file_1.docx]

**Supplementary Text S1. Detailed software commands, parameters, and database version used in this research.**

To ensure reproducibility, we provide the exact command lines and parameter settings for the bioinformatic tools used in this genomic analysis. Unless otherwise specified, default parameters were applied for all software.

**1. Taxonomic Classification**

- **OrthoANI:**

• **Software**: OAT (OrthoANI Tool) v0.93.1

• **Command**: java -jar OAT_cmd.jar -fasta1 [genome1.fasta] -fasta2 [genome2.fasta]

• **Parameters**: Default settings were used for calculating Average Nucleotide Identity.

- **dDDH (digital DNA-DNA Hybridization):**

• **Software**: GGDC (Genome-to-Genome Distance Calculator) v3.0

• **Server Access**: https://ggdc.dsmz.de/

• **Parameters**: Default settings were used for calculating distance.

**2. Pan-genome Analysis**

• **Software**: PPanGGOLiN (v2.1.0)

• **Command**: ppanggolin all --fasta [genome_fasta_list] --anno [gff_list] --output [output_dir] --rarefaction --cpu [num_threads] --kingdom bacteria

• **Parameters**: Parameters not specified above were set as default.

**3. Whole-Genome Phylogenetic Analysis**

• **Software**: CVTree3 (Standalone local version)

• **Command**: cvtree -i [proteome_list] -G [input_folder] -k ‘5,6,7’

• Parameters: Parameters not specified above were set as default.

**4. Virulence Factor Identification**

• **Software**: BLAST+ (v2.13.0)

• **Database**: Virulence Factor Database (http://www.mgc.ac.cn/VFs/)

• **Method**: Virulence factor genes were identified using BLASTP searches against the VFDB core dataset (Set A).

• **Parameters**: E-value ≤ 1e−5, Identity ≥ 70%

**5. Insertion Sequence Prediction**

• **Software**: ISEScan (v1.7.2.3)

• **Command**: isescan.py --seqfile [genome.fasta] --output [result_dir] --nthread [num_threads]

• **Parameters**: Parameters not specified above were set as default.

**6. Secretion System Prediction**

• Software: DeepSecE (v0.1.2)

• Command: python3 predict.py --fasta_path [proteins.faa] --model_location [model_path] --data_dir [data_dir] --out_dir [output_dir]

• Parameters: Parameters not specified above were set as default.

**7. Antibiotic Resistance Gene Screening**

• **Software**: ABRicate (v0.9.8)

• **Database**: NCBI Bacterial Antimicrobial Resistance Reference Gene Database, ResFinder, Comprehensive Antibiotic Resistance Database (CARD)

**• Command**: abricate [genome.fasta] > [output_path]

• Parameters: Parameters not specified above were set as default.

**8. CAZyme Annotation**

• **Software**: run_dbcan (Standalone version from run_dbcan (v4.1.4) suite)

• **Command**: run_dbcan [input_file_path] protein --out_dir [output_dir] --db_dir [db_database_dir]

• **Parameters**: Parameters not specified above were set as default.

**9. Circular Genome Visualization**

• **Software**: Circos (v0.69-9)

• **Usage**: Circular representation of genomic features (Figure 1 & S1).

• **Parameters**: Tracks configured for forward/reverse CDS, GC content, and GC skew (Window size: 1,000 bp).

**10. Network Analysis and Visualization**

• **Software**: Gephi (v0.10.1)

• **Layout**: ForceAtlas2 algorithm (Scaling: 8,000, Gravity: 4.0, edge weight influence: 1.3) was used to spatialise the gene co-occurrence network.

• **Community Detection**: Modularity class algorithm (Resolution: 1.0) was applied to identify gene clusters.

• **Centrality Metrics**: Degree centrality and Betweenness centrality were calculated using the internal statistics module.

**11. Phylogenetic Tree Visualization**

• **Software**: GraPhlAn (v1.1.3)

• **Command**: Annotation - graphlan_annotate.py --annot [annotation.txt] [input.tree] [output.xml]

Rendering - graphlan.py [output.xml] [image.png] --dpi 300

**12. Machine Learning Analysis**

• **Software:** Orange3 (v3.38.1)

• **Input**: CAZyme matrix (CSV format)

• **Preprocessing**: Missing values were handled using the 'Impute' widget (default), and the target variable (pirA/B present) was defined using the 'Select Columns' widget.

• **Modeling** **& Validation**: 10-fold Cross-Validation was performed using the 'Test and Score' widget.

• **Algorithms**: Random Forest, SVM, Logistic Regression, and Gradient Boosting were executed using default hyperparameters provided by the Orange environment to prevent overfitting.
